# RefCap: image captioning with referent objects attributes

**DOI:** 10.1038/s41598-023-48916-6

**Published:** 2023-12-07

**Authors:** Seokmok Park, Joonki Paik

**Affiliations:** 1 Department of Image, Chung-Ang University, 84 Heukseok-ro, Seoul, 06974 Republic of South Korea; 2 Department of Artificial Intelligence, Chung-Ang University, 84 Heukseok-ro, Seoul, 06974 Republic of South Korea

**Keywords:** Electrical and electronic engineering, Computer science

## Abstract

In recent years, significant progress has been made in visual-linguistic multi-modality research, leading to advancements in visual comprehension and its applications in computer vision tasks. One fundamental task in visual-linguistic understanding is image captioning, which involves generating human-understandable textual descriptions given an input image. This paper introduces a referring expression image captioning model that incorporates the supervision of interesting objects. Our model utilizes user-specified object keywords as a prefix to generate specific captions that are relevant to the target object. The model consists of three modules including: (i) visual grounding, (ii) referring object selection, and (iii) image captioning modules. To evaluate its performance, we conducted experiments on the RefCOCO and COCO captioning datasets. The experimental results demonstrate that our proposed method effectively generates meaningful captions aligned with users’ specific interests.

## Introduction

Visual understanding, which is analogous to human visual perception, is a major research topic in computer vision research. An essential aspect of this understanding involves resolving the intricate relationships between visual information and textual associations. Image captioning is a fundamental task in visual understanding, where human-comprehensible textual information is derived from analyzing visual data. Current image captioning research primarily focuses on attention mechanisms, commonly employing region proposal networks to obtain region features for attention modeling. In addition, the field of visual-linguistic multi-modality has exhibited significant advancements in image captioning, with notable contributions such as Contrastive Language-Image Pre-Training (CLIP)^1^. CLIP is designed to learn contrastive loss from extensive datasets, understanding visual features and textual descriptions, thereby establishing meaningful and correlated visual-text representations.

Dense captioning, a subcategory of image captioning, predicts diverse captions from a given input image, instead of being limited to specific caption outcomes^2,3^. By selecting various objects in the image, dense captioning can capture aspects of multiple situations. However, even though there are many dense captions available, the difficulty lies in choosing truly meaningful captions. In this paper, we introduce a novel approach called RefCap (Image Captioning with Referent Objects Attributes) for generating meaningful captions that align with user preferences in referring expression image captioning. This approach enhances visual understanding by incorporating object relation descriptors. In the proposed RefCap model, after a region proposal network detects various objects, our approach selectively focuses on the related objects based on user input prompts. This enables RefCap to predict specific caption outcomes that correspond to the objects and their referring expressions provided by the user, thus providing a more targeted caption generation instead of a range of possible outcomes.

Visual grounding (VG), also referred to as referring expression comprehension, is an intriguing research area in the field of computer vision^4^. VG methods aim to identify objects in an image that correspond to a user’s query. For example, if the user inputs a query such as “the man on the right,” the visual grounding module identifies and highlights the specified referent, and then the captioning module generates the corresponding expression. In our RefCap model, we utilize the localized objects obtained from the VG task’s results and establish their relationships.

**Figure 1:**
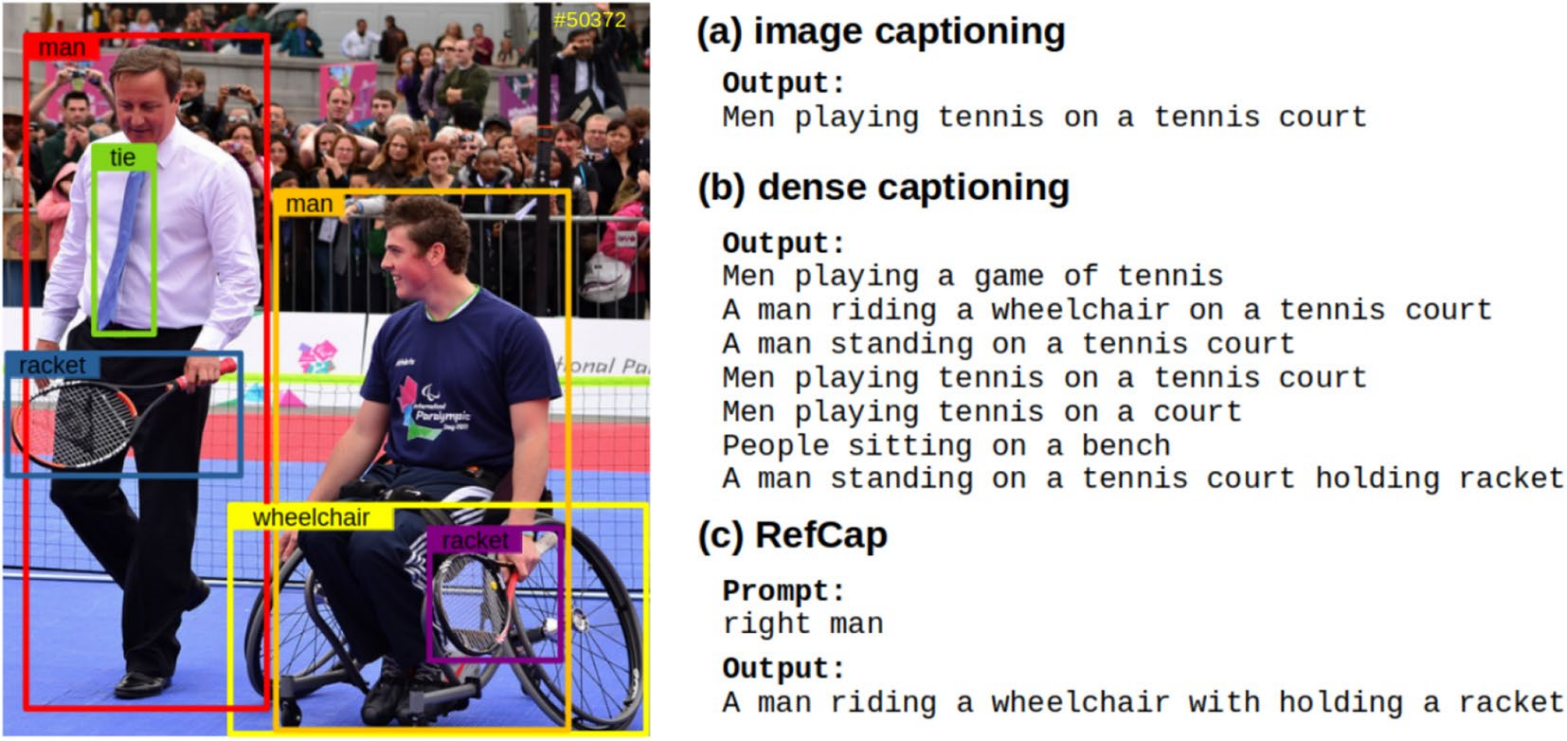
Comparison between RefCap and other approaches: (**a**) image captioning, (**b**) dense captioning, and (**c**) RefCap. The given image is the sampled from the COCO2014 train dataset. The $\#$ means the image index of the dataset.

**Figure 2:**
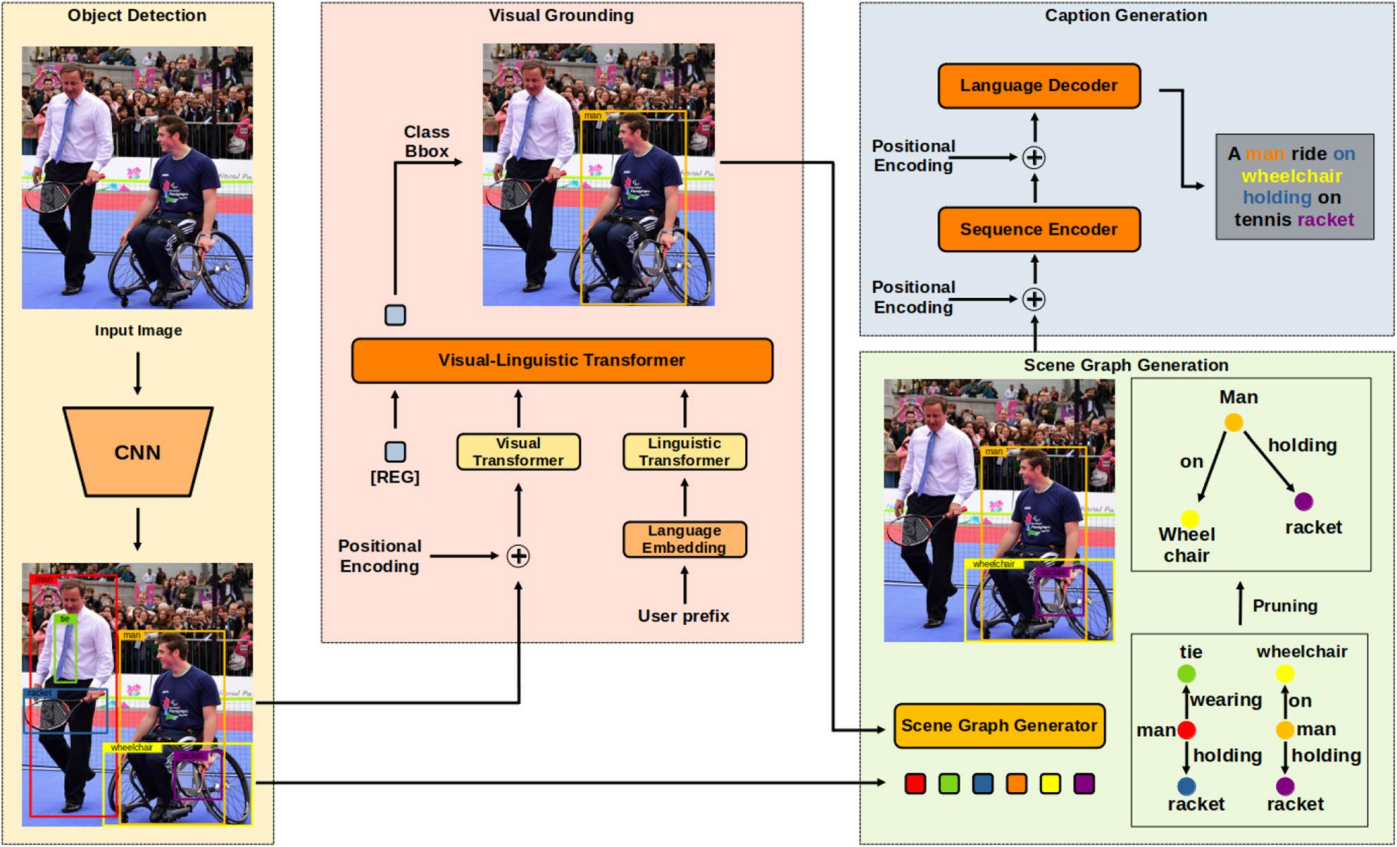
The RefCap model generates a caption for the object specified by the user prefix in the same image as shown in Figure  1.

Figure  2 illustrates the pipeline of our RefCap model, which combines four main computer vision algorithms: object detection, visual grounding, scene graph generation, and image caption generation. A description of each pipeline network’s role is provided in the “Proposed Method” section. Compared to other tasks of visual understanding, image captioning, and dense captioning, our RefCap model has the following differences, as shown in Figure 1. The image captioning task derives the most descriptive single textual information about a given image. Compared to image captioning, the dense captioning task can express more diverse information in the image. However our RefCap model, the user first selects the object of interest in a given image and then derives a corresponding representation of that object. The RefCap model has the following steps:

1. The word-level encoder encodes the user prompt, which is then passed to the Transformer along with the output of the object detection task.
2. The VG task provides localization information about the target query, which is then used to construct object-level relations with a given object.
3. Finally, we perform image captioning (IC) using the constructed relations to derive textual information about the object relationships.

Compared to other visual understanding tasks, such as image captioning and dense captioning, our RefCap model has the following key differences:

- Image captioning generates a single textual description of a given image.
- Dense captioning generates multiple textual descriptions of different objects in a given image.
- RefCap generates a textual description of a user-specified object in a given image.

## Related work

In the past few years, the field of image captioning and visual-linguistic multi-modality research has exhibited significant advancement. In this section, we briefly review related literature for image captioning, visual-linguistic model, and referring expression with discussion about the limitations of existing approaches.

### Image captioning

In the early stages of image captioning, approaches primarily relied on pre-defined caption templates or matching image regions to textual descriptions. Hodosh et al. introduced methods such as template matching and retrieval-based image captioning^5^. Gan et al. used the object detection results as input to the LSTM to generate the caption^6^. However, these methods faced limitations in terms of flexibility and the ability to generate diverse, contextually relevant captions.

With the advent of deep learning, the focus shifted towards using neural networks for image captioning. A representative work is the Show and Tell model by Vinyals et al. , which introduced an encoder-decoder framework using a convolutional neural network (CNN) as an image encoder and a recurrent neural network (RNN) as a caption generator^7^. This approach significantly improved the quality of generated captions by learning rich visual representations and capturing temporal dependencies in language.

Recently, researchers have also studied temporal information-based caption generation approaches. For example, Tu et al. investigated how to derive a representative caption that expresses a video’s temporal causal relationship and how to find changes in multiple images^8,9^.

### Visual-linguistic models

To enhance the visual-linguistic understanding, recent research efforts have explored incorporating attention mechanisms into image captioning models. Xu et al. proposed the attention mechanism, allowing the model to selectively focus on different image regions while generating captions^10^. Similarly, Lu et al. ^11^ introduced the adaptive attention model, which dynamically adjusted the attention weights based on the input image and generated caption. These attention-based models achieved better alignment between the generated words and visual content, leading to improved caption quality^11,12^.

Some studies integrate the extensive prior knowledge of visual-linguistic models, such as ClipCap^13^ and SMALLCAP^14^, which combine CLIP and GPT-2 with vast amounts of pre-trained models to present lightweight-training models with only minimal fine-tuning for image captioning.

### Referring expression generation

In the field of visual understanding research, the generation of referring expressions has gained increasing attention. In this context, Kazemzadeh et al. presented the RefCOCO dataset, which contains referring expressions for objects in images^15^. Mao et al. proposed a language-guided attention model to generate referring expressions, which makes the model attend to relevant image regions described in the expression^16^. More recently, referring expressions image captioning has become a crucial research topic in the visual-linguistic multi-modality research field. Hu et al. proposed an end-to-end referring expression image captioning model that incorporated explicit supervision of referred objects^17^. In their model, user-specified object keywords are used as a prefix to generate specific captions focused on the target object to improve alignment between the referred object and the generated captions.

### Visual scene graph generation

The goal of the visual scene graph generation (SGG) task is to detect the relationships between objects. The representative dataset is Visual Genome which is presented by Ranjay et al. ^18^. The Visual Genome dataset provides localized bounding boxes for objects, along with their attributes such as color, size, location, and more. Furthermore, objects are cross-connected through the relationships. Understanding contextual relationships between objects in the image and its corresponding language is useful for downstream visual-linguistic understanding tasks. In the SGG task, the encoder-decoder method is generally utilized. Xiao et al. generated the graph using both spatial and semantic attention modules and its fusion^19^. Cong et al. proposed a one-stage approach that can predict the relations directly using a triplet decoder, and they also provide abundant demonstrations^20^.

There is also a study that has attempted to image captioning through the SGG task. Yang et al. ^21^ presented a method for image captioning through the SGG task using a homogeneous network to convert a scene graph into a caption^21^. They used a scene graph to represent objects in the image and generated a caption via an encoder-decoder structure.

## Proposed method

In this section, we present an image captioning model called RefCap using referent object relationships. As shown in Figure 2, RefCap requires user prompts to initiate the captioning process. Subsequently, it employs Visual Selection (VS) and Image Captioning (IC) tasks to derive a textual description of the selected object and its corresponding referent. To gain a better understanding of RefCap’s functionality, we provide a detailed explanation in the following subsections.

### Visual grounding

For the visual grounding task, both visual and linguistic features are used to compute embedding vectors as input. Given an image as input, the visual branch is composed of a stack of 6-encoder layers. Each encoder layer of the visual branch includes a multi-head self-attention layer and feed-forward network (FFN). Positional encoding is then added at each encoder layer. Meanwhile, the linguistic branch utilizes a 12-encoder layer with a pre-trained BERT model. A [CLS] token is appended at the beginning, and a [SEP] token is appended at the end of each token. Subsequently, each token is used as input for the linguistic transformer. To merge these visual-linguistic tokens, a linear projection is applied to them with the same dimension, and a learnable regression token [REG] is added for bounding box prediction. The visual-linguistic fusion tokens are then fed into a visual-linguistic transformer with six encoder layers. To configure a loss function, we sum the differences between predicted and ground-truth boxes across all stages to calculate the total loss.

1 $$\begin{aligned} L = \alpha _1L_1 + \alpha _gL_{giou}, \end{aligned}$$

where $\alpha _1$ and $\alpha _g$ are hyper-parameters. $L_1$ is the $\ell _1$ loss and $L_{giou}$ is the generalized intersection over union (GIoU) loss proposed by Rezatofighi et al. ^22^. As a result, the bounding box corresponding to the user prefix is predicted.

### Referent objects selection

Following the Visual Grounding (VG) task, our model establishes relationships between referent objects. To construct these relationships, we require additional object locations. By utilizing a CNN-based object detection algorithm, we can easily obtain localized objects. The image features are then linearized to form the input vector, and a subset of these features is fed into the transformer encoder layer.

For every possible relationship between the objects, we compute the probability of their relationships. Considering that there are $m(m-1)$ potential relationships among *m* objects, we generate $m(m-1)$ pairwise features. Thus, the relationships between the *i*-th subject and the *j*-th object can be denoted as $R_{i\rightarrow {j}}\in R$, where $i \ne j$. These relationships form a directed graph represented by a collection of subject-predicate-object triplets.

The triplet prediction can be expressed as:

2 $$\begin{aligned} \hat{R} = <\hat{y}_{\text {sub}}, \hat{c}_{\text {pred}}, \hat{y}_{\text {obj}}>, \end{aligned}$$

where each subject and object contain class and bounding box labels denoted as $\hat{y} = <\hat{c}, \hat{b}>$. Using the ground truth triplet $R = <y_{\text {sub}}, c_{\text {pred}}, y_{\text {obj}}>$, the triplet cost is computed using the cost function $c_m$ introduced in RelTR^20^.

3 $$\begin{aligned} c_{tri}=c_m(\hat{y}_{sub},y_{sub})+c_m(\hat{c}_{pred},c_{pred})+c_m(\hat{y}_{obj},y_{obj}). \end{aligned}$$

Given the triplet cost $c_{tri}$, we can get the triple loss $L_{tri}$ as:

4 $$\begin{aligned} L_{tri}=L_{sub} + L_{obj} + L_{pred}, \end{aligned}$$

where *L* is the cross-entropy loss between the predicted class and the ground truth class.

As following steps from RelTR, we can get pruned relationships such as <Obj-relation-BG> and <Obj-no relation-Obj>. These dense object relationships obtain abundant descriptions between subject-object relations. However, unnecessary relations still follow. Therefore we need to prune some edges for remaining meaningful relations. For remaining referent objects relationships, we apply some criteria:

1. The initial object $y_{init}$ from the VG task is root node.
2. The duplicated relationship must not be included.
3. The subject which is predicted from subject $y_{init}$ or $y_{obj}$ as object previous step, can have another relationship.
4. The object which is predicted from object $y_{init}$ or $y_{sub}$ as subject previous step, can have another relationship.
5. Both subject and object can each have multiple relationships, unless the above paragraph is contradicted.

Finally, we perform image captioning (IC) using the constructed relations to derive textual information about the object relationships.

### Image captioning

The final step of our model is to generate the target caption result by incorporating the structure derived from the selected referent objects. Before entering the caption step, we concatenate the output of SGG and VG tasks. However predicted triplet embeddings $\hat{R}=<\hat{y}_{sub},\hat{c}_{pred},\hat{y}_{obj}>$ and visual features contain different information and lengths. Therefore, we need to unify both triplet embeddings and visual features as the same dimension features. These concatenated features enter the encoder of the transformer network^23^. The Encoder layer is composed of a stack of 6-encoder layers, and each encoder layer includes a multi-head self-attention layer and FFN similar to our VG task. As following the transformer^23^, the attention can be calculated by:

5 $${\text{Attention}}(\textbf{Q},\textbf{K},\textbf{V})={\text {softmax}} \left( \frac{\textbf{Q}\textbf{K}^{\textrm{T}}}{\sqrt{d_K}}\right) \textbf{V}, $$

where $\textbf{Q},\textbf{K},\textbf{V}$ is query, key, value of attention module, $d_K$ is dimension of model. And the multi-headed attention is also calculated by:

6 $$\begin{aligned} \text {Multihead}(\textbf{Q},\textbf{K},\textbf{V})=\text {Concat}(h_1,h_2,...,h_n)W^\text {o}, \end{aligned}$$

where $h_i=\text {Attention}(\textbf{Q}W^q_i,\textbf{K}W^k_i,\textbf{V}W^v_i)$ and the projections *W* is parameter matrices of each head. The encoded features are passed to the linguistic decoder for captioning. The linguistic decoder is also composed of a stack of 6-decoder layers. By decoding the features, we finally obtain a caption that corresponds to the user input object.

The objective function for image captioning consists of two terms: cross-entropy loss and Self-Critical Sequence Training (SCST) loss^24^. The cross-entropy loss is defined as follows:

7 $$\begin{aligned} L_{CE}=-{\sum ^T_i \log (p_\theta (t^{*}_t|y^{*}_1,...,y^{*}_{t-1}))}, \end{aligned}$$

where $y^*_t$ is the ground-truth target for all sequences, and $\theta$ is the model parameters. The SCST loss minimizes the negative expectation of the CIDEr score, which is a metric for evaluating the quality of image captions:

8 $$\begin{aligned} L_{SCST}=-E_{y_{1:T}\sim {}p_\theta }[r(y_{1:T})], \end{aligned}$$

where *r* denotes the CIDEr fraction function. The gradient of the SCST loss can be approximated as:

9 $$\begin{aligned} \nabla _\theta L_{SCST}\approx -(r(y_{1:T})-r(\hat{y}_{1:T}))\nabla _\theta \log p_\theta (y_{1:T}), \end{aligned}$$

where $r(y_{1:T})$ is the CIDEr score of the sampled caption and $r(\hat{y}_{1:T})$ is the CIDEr score of the greedy decoding of the model.

## Experimental results

### Implementation details

In our experimental results, we present a comprehensive evaluation of each module, incorporating both quantitative and qualitative assessments. Our RefCap model consists of four main modules, namely:

(i) Object detection: This module enables the system to search for relevant visual content based on user queries.
(ii) Visual grounding: The visual grounding module aims to establish a connection between textual queries and specific objects or regions in the visual content.
(iii) Scene graph generation: This module generates a structured representation of the relationships between objects in the scene, capturing their interactions and contextual information.
(iv) Image captioning: The image captioning module generates descriptive captions that accurately convey the content and context of the visual input.

Each of these modules plays a crucial role in our RefCap model, and we provide detailed descriptions and evaluations for each module in the following sections.

#### Object detection

For object detection, we utilize a Faster R-CNN model^25^ pretrained on the ImageNet dataset, with ResNet-101^26^ as the backbone architecture. This model is then fine-tuned on the Visual Genome dataset to perform visual grounding and scene graph generation tasks.

To reduce the dimensionality of the object features, which initially yield a 2048-dimensional feature vector, we apply dimensionality reduction to obtain a dimension of $d_K=512$. This reduction is followed by a ReLU activation and a dropout layer to enhance the model’s performance. During training, we employ the SGD optimizer with an initial learning rate of $1\times 10^{-2}$.

**Table 1:** Comparison of RefCap to state-of-the-art methods on the ReferItGame, RefCOCO, and RefCOCO+ datasets.

Models	ReferItGame	RefCOCO	RefCOCO+
MMI^16^	–	71.38	59.17
VC^27^	30.92	73.35	58.42
MAttNet^28^	29.04	**82.30**	72.63
SSG^29^	55.12	76.63	59.12
RCCF^30^	64.21	81.14	69.87
RefCap	**70.21**	82.23	**73.08**

Signifiacne values are in bold.

#### Visual grounding

To evaluate the Visual Grounding task, we conduct experiments on two datasets: ReferItGame^15^ and RefCOCO^31^. These datasets consist of images that contain objects referred to in the referring expressions. Each object may have one or multiple referring expressions associated with it.

We split each dataset into three subsets: a 70% training set, a 20% test set, and a 10% validation set. The input image size is standardized to $640 \times 640$, and the maximum expression length is set to 10 tokens, including the [CLS] and [SEP] tokens. The shorter maximum expression length is chosen because the inference process only requires the keywords related to the target object.

These evaluation setups allow us to assess the performance of the Visual Grounding task on different datasets and validate the effectiveness of our approach.

#### Scene graph generation

The Visual Genome dataset is used for evaluating Scene Graph Generation. The Visual Genome dataset consists of 108K images with 34K object categories, 68K attribute categories, and 42K relationship categories. We select the most frequent 150 object categories and 50 relationship categories. The attribute categories are omitted by merging with relationship categories. Thus, each image has object and relationship (with attributes) categories in the scene graph. During inference, the criteria are applied aforementioned in the referent object selection section for pruning the unrelated relationships.

#### Image captioning

For the image captioning task, the commonly used COCO Entities dataset^32^ was used. The dataset contains diverse caption annotations with an abundant combination of objects and their bounding boxes. Thus employing these datasets by RefCap which builds a sub-graph makes sense.

### Quantitative evaluation

We first evaluate the visual grounding task of RefCap on the ReferItGame^15^, RefCOCO^31^, and RefCOCO+^31^ datasets, comparing it to other state-of-the-art methods including Maximum Mutual Information (MMI)^16^, Variational Context (VC)^27^, Modular Attention Network (MAttNet)^28^, Single-Stage Grounding (SSG)^29^, and Real-time Cross-modality Correlation Filtering (RCCF)^30^. Note that the accuracy of the RefCOCO and RefCOCO+ datasets is based on TestA only. Table 1 shows that our RefCap model is competitive with other state-of-the-art methods.

**Table 2:** Evaluation with four metric.

PredCls		PhrCls		SGGen		SGGen+	
R@50	R@100	R@50	R@100	R@50	R@100	R@50	R@100
48.9	52.3	28.9	30.2	12.1	13.4	27.8	36.2

As following yang et al. ^33^, Predicate Classification (PredCls) means the performance for recognizing the relation between two objects given the GT locations, Phrase Classification (PhrCls) means the performance for recognizing two object categories and their relation given the GT locations, Scene Graph Generation (SGGen) means the performance for detecting objects and recognizing the relations between object pairs, and Comprehensive Scene Graph Generation (SGGen+) not only considers the triplets but also the singleton (object and predicate).

We also evaluate our visual scene graph generation of RefCap with grounded objects on the Visual Genome dataset. Our desired output of the scene graph is the sub-graph of the entire graph. This means the result doesn’t need to include the entire relationship. Thus we aim that how the sub-graph represents well about target object and the output includes ground truth. We adopt the four metrics (PredCls, PhrCls, SGGen, SGGen+) for evaluating our scene graph generation which is presented by Yang et al. ^33^ with our insight. We modified the ground truth data to reference the target object. Each image contains multiple target objects and its referent relationship. Thus we just compare the relation to the target object with modified ground truth. The performance of generating sub-graph by RefCap is shown in Table 2.

We finally evaluate our image captioning task. We employ conventional metrics (BLEU^34^, METEOR^35^, ROUGE^36^, and CIDEr^37^) to measure the quality of the predicted captions on the COCO Entities dataset. Table 3 shows the results of evaluating the predicted caption on COCO Entities.

**Table 3:** Evaluation of RefCap on the image captioning task on the COCO Entities dataset.

Models	BLEU-4	METEOR	ROUGE	CIDEr
SCST^24^	25.3	25.7	50.1	131.4
Up-Down^38^	25.5	26.8	53.2	137.1
ClipCap^13^	33.5	30.4	–	124.1
GRIT^39^	**38.2**	**30.3**	55.7	142.9
RefCap	33.2	29.7	**56.2**	**143.7**

Signifiacne values are in bold.

### Qualitative evaluation

**Figure 3:**
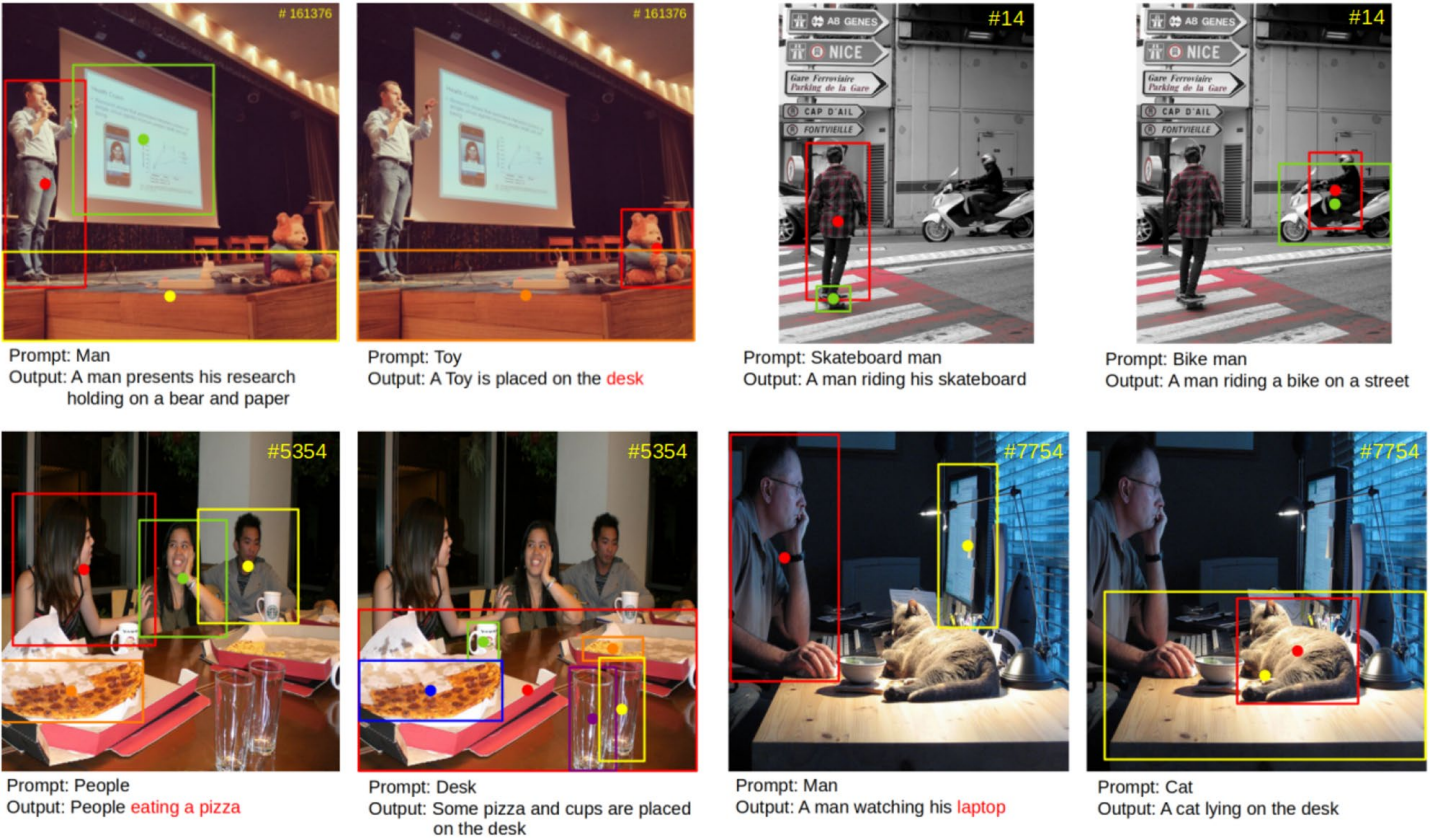
Some examples of RefCap model. The given images are selected from the COCO2014 test dataset. The $\#$ means the image index of the dataset. Incorrect caption results are highlighted in red.

Figure 3. shows the examples of our entire RefCap model. The few keywords are typed as input by the user, RefCap detects the corresponding object, builds a relationship with related objects, and draws its caption result. Unlike traditional caption methods, RefCap shows the caption results for the user’s desired target. Our RefCap can provide caption results, not only in images with a single object but also in images with multiple objects.

### Ablation study

**Figure 4:**
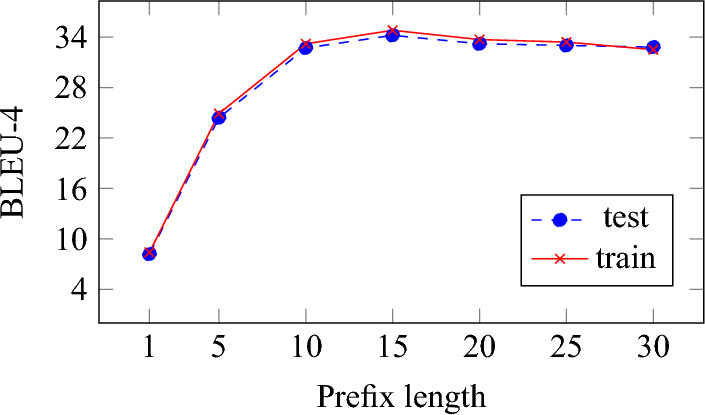
Effect of prefix length on the image captioning performance of RefCap.

In this section, we analyze the impact of each hyperparameter on the model for each module. First, we explored the effect of the prefix length on the visual grounding (VG) task. As summarized in Fig. 4, the performance tends to improve as the prefix length increases. However, continually increasing the prefix length slows down processing due to the increased parameters of the model. Therefore, RefCap uses a prefix length of 15, which balances performance and processing time.

In a separate experiment, we examined how the scene graph generator (SGG) affects the performance of caption generation. Table 4 shows the accuracy of different combinations of subject, predicate, and object. The results indicate that using all three components achieved the best outcome. As you can see, the combination of object and predicate is superior to subject and object alone, because it is difficult to represent the target’s properties with class alone.

**Table 4:** Evaluation of scene graph generation on the Visual Genome dataset.

Models	BLEU-4	METEOR	ROUGE	CIDEr
Object + subject	6.2	12.8	24.2	45.9
Object + predicate	14.8	20.5	34.1	74.4
Object + subject + predicate	**18.3**	**22.4**	**42.1**	**86.6**

Signifiacne values are in bold.

## Research plan

In this paper, we introduce a novel image captioning model, RefCap, which leverages referent object attributes to generate more specific and tailored captions. However, our model has several limitations. First, it consists of a combination of several pipeline networks, which makes it complex and sensitive to the performance of each individual network. To address this, we plan to develop an end-to-end model in our future work. We look forward to sharing our progress in future publications.

## Discussion and conclusion

The main idea of the paper is to predict a meaningful caption from a selected user prefix. By exploring object relationships for image captioning, our method can more accurately and concretely predict the caption results. As a result, the user of our method can get the more satisfying result that corresponds to his prefix. We also demonstrated quantitative evaluation and qualitative evaluation. As a quantitative evaluation, we experiment with various datasets for each module. Both quantitative and qualitative evaluations yielded gratifying results. Moreover, our RefCap can provide multiple caption results from a single image based on user input. We hope the utilization of this convergence of the object detection and image captioning tasks, would provide insight into the future of computer vision and multimodality research.

## Data Availability

All data generated or analyzed in this study are included in this published article. The training and testing datasets used in this study are publicly available and have been cited in accordance with research rules. Detailed descriptions of the datasets and their citations can be found in the “Experimental results” section of the paper. For instance, the ReferItGame, RefCOCO, and RefCOCO+ dataset’s training set can be downloaded from https://github.com/lichengunc/refer. Furthermore, The COCO2014 dataset and Visual Genome dataset’s training set can be accessed via https://cocodataset.org, https://homes.cs.washington.edu/ ranjay/visualgenome/index.html, respectively. The testing set of the COCO Entities dataset can be downloaded from https://github.com/aimagelab/show-control-and-tell, respectively.
